# P-220. Real-World Effectiveness of Fecal Microbiota, live-jslm for the Prevention of Recurrent *Clostridioides difficile* Infection

**DOI:** 10.1093/ofid/ofae631.424

**Published:** 2025-01-29

**Authors:** Richard L Hengel, Sujatha Krishnan, Timothy E Ritter, Jonathan A Rosenberg, Kathy A Baker, Lucinda J Van Anglen, Kelly E Hanna, Amy Guo, Mielad Moosapanah, Sanghyuk Seo, Kevin W Garey

**Affiliations:** Atlanta ID Group, Atlanta, Georgia; DFW Infectious Diseases, PLLC, Frisco, Texas; GI Alliance, Southlake, Texas; Medical Director of Research GI Alliance of Illinois, Highland Park, Illinois; Texas Christian University, Fort Worth, Texas; Healix Infusion Therapy, LLC, Sugar Land, Texas; Healix Infusion Therapy, LLC, Sugar Land, Texas; Ferring Pharmaceuticals, parsippany, New Jersey; Ferring Pharmaceuticals, Inc., Parsippany, New Jersey; Ferring Pharmaceuticals, Inc., Parsippany, New Jersey; University of Houston, Houston, TX

## Abstract

**Background:**

Fecal microbiota, live-jslm (RBL) is a live biotherapeutic product approved by the FDA in November 2022 for the prevention of recurrence of *Clostridioides difficile* infection (rCDI) in adults. It is a rectally administered, pre-packaged single dose microbiome therapy, demonstrated to be safe and efficacious in clinical trials. There are few real-world studies of RBL use in routine clinical practice. This study reports effectiveness of RBL in an outpatient real-world setting.

**Figure:**
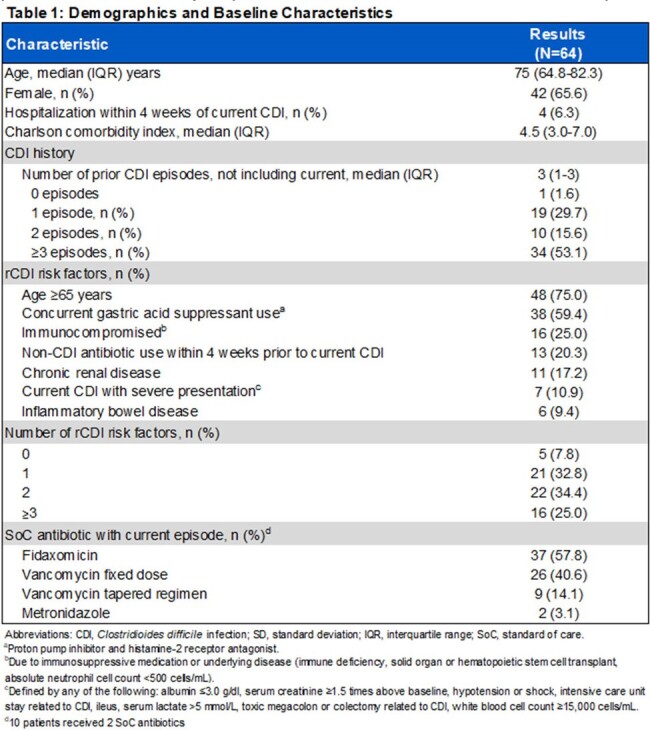

**Methods:**

Medical records of patients who were administered RBL within physician offices nationally were reviewed from February 2023 to March 2024. Data included patient demographics, comorbidities, rCDI history, risk factors and treatments, including standard of care antibiotic (SoC). Recurrence, defined as any occurrence of diarrhea with 3 liquid bowl movements or more within 24 hours, was assessed at 8 weeks post-RBL administration.

**Results:**

RBL was administered to 64 patients from 32 physician offices. Patient characteristics are shown in Table 1. Patients had a median age of 75 (IQR: 64.8-82.3) years and were predominantly female (65.6%). Patients had multiple comorbidities (Charlson score 4.5; IQR: 3-7) and 59.4% had 2 or more rCDI risk factors. The most common risk factors were age ≥65 years (75%) and concurrent gastric acid suppressant use (59.4%). The median number of prior CDI episodes was 3 (IQR: 1-3). Fidaxomicin (57.8%) was the most utilized SoC antibiotic for treatment of CDI prior to administration of RBL. There were no adverse events reported with administration of RBL other than minor leakage during administration in 4 (6.2%). Eight-week follow-up was completed in 59 patients, of which 25% (n=15) experienced a recurrence of CDI.

**Conclusion:**

This real-world study demonstrated that RBL treatment to prevent recurrence of CDI was safe and effective in routine clinical practice.

**Disclosures:**

**Richard L. Hengel, MD**, Gilead: Grant/Research Support **Timothy E. Ritter, MD**, Abbvie: Advisor/Consultant|Boehringer Ingelheim: Advisor/Consultant|Bristol Myers Squibb/Celgene: Advisor/Consultant|Eli Lilly: Advisor/Consultant|Ferring: Advisor/Consultant|Ferring: Data Adjudication Committee|Genetech/Roche: Advisor/Consultant|Gilead: Advisor/Consultant|Intercept: Advisor/Consultant|Iterative Health: Advisor/Consultant|Janssen: Advisor/Consultant|Merck: Advisor/Consultant|Pfizer: Advisor/Consultant|Rebiotix: Data Adjudication Committee|Sanofi: Advisor/Consultant|Takeda Pharmaceuticals: Advisor/Consultant **Jonathan A. Rosenberg, MD**, Aimmune: Advisor/Consultant|Ferring Pharmaceuticals: Advisor/Consultant **Lucinda J. Van Anglen, PharmD**, Cumberland Pharmaceuticals: Grant/Research Support|Ferring Pharmaceuticals: Grant/Research Support|Novartis Pharmaceuticals: Grant/Research Support|Takeda Pharmaceuticals: Grant/Research Support **Amy Guo, PhD**, Ferring Pharmaceuticals, Inc.: Employee **Mielad Moosapanah, PharmD**, Ferring Pharmaceuticals: Employee **Sanghyuk Seo, PharmD, MS**, Ferring Pharmaceuticals, Inc.: Employee **Kevin W. Garey, PharmD, MS**, Acurx: Grant/Research Support

